# Encapsulating Peritoneal Sclerosis – A rare and serious complication of peritoneal dialysis: Case series

**Published:** 2014

**Authors:** O Mihalache, C Bugă, H Doran, E Catrina, F Bobircă, T Pătrașcu

**Affiliations:** * ”Carol Davila” University of Medicine and Pharmacy, Bucharest, Romania; ** Surgical Clinic I, “Juvara” Clinical Hospital; “Dr. I. Cantacuzino” Hospital, Bucharest, Romania

**Keywords:** peritoneal dialysis, encapsulated peritoneal sclerosis, chronic kidney disease

## Abstract

**Introduction.** Encapsulating peritoneal sclerosis is a pathological entity mainly associated with peritoneal dialysis (PD). The clinical syndrome is characterized by various degrees of intestinal obstruction due to thickening, sclerosis and calcification of peritoneum resulting in the encapsulation and cocooning of the bowel. It is a rare but potentially devastating complication associated with a considerable morbidity and mortality.

**Materials and methods.** Cases of encapsulating peritoneal sclerosis (EPS), diagnosed in the Surgical Clinic of “Cantacuzino” Hospital, between 2007 and 2014 were retrospectively reviewed. During this interval, 432 surgical interventions related to peritoneal dialysis were performed: 306 peritoneal access interventions and 124 complications, of which 15 patients with EPS.

**Results.** In all but two cases, the EPS diagnostic was established at the time of the surgical intervention addressed to other complication or pathology. Moreover, in 2 of the 15 patients the diagnostic was established approximately 5 months after PD was discontinued, and, in one of these patients at the time of the extraction of the dialysis catheter. 12 of 15 patients were diabetic. Most patients had a history of multiple peritonitis episodes. All the patients required the passing from peritoneal dialysis to hemodialysis. There were 4 deaths (26,6%) of which one was around two months from the diagnosis.

**Conclusions.** The timely diagnosis of the condition and the appropriate phase-specific treatment is of utmost importance in EPS. In advanced stages, the surgical intervention performed by a well-trained team could achieve good long-term results.

## Introduction

Encapsulating peritoneal sclerosis (EPS) is a severe complication of the peritoneal dialysis, described for the first time in 1980 by Gandhi and others. It is defined by the Ad Hoc Committee of International Society of Peritoneal Dialysis as a clinical syndrome characterized by a persisting intestinal obstruction, intermittent or recurrent, associated with peritoneal thickening, sclerosis, calcification or encapsulation with or without the presence of inflammation markers [**1**]. The reported incidence ranges between 2.1% and 19.4% in patients under peritoneal dialysis (PD) of 5-8 years old. Australia reports an incidence of 6.4% at 5 years and 19.4% at 8 years, respectively, and Japan reports 2.1% and 5.9%, respectively [**4**]. According to the Scottish Renal Registry, between 2000 and 2007, EPS had an incidence of 3.5% after 3-4 years from the peritoneal dialysis and of 8% after 4-5 years [**5**]. The encapsulating peritoneal sclerosis may occur and progress even after the PD is discontinued and the patient is transferred to haemodialysis or after performing a renal transplant [**4**]. Although the incidence is not large, by comparison to other complications, EPS represents the most severe complication of PD, due to the high mortality rate associated to it, as the said mortality reaches a value of around 50% usually in 12 months from the diagnostic and reaching a value as high as 100% in patients who have been on peritoneal dialysis for over 15 years [**4**-**6**]. The etiology and pathogenesis of EPS are not yet elucidated. A series of factors related or not to PD contribute to the alteration of the normal physiology of the peritoneum. The most important risk factor related to PD is its long duration, to which the following can be added: use of bioincompatible solutions, inflammation caused by repeated peritonitis, exposure to miscellaneous chemical agents (chlorhexidine), genetic factors or other PD unrelated factors. No predictive risk factors to prevent the occurrence of EPS were identified [**2**,**16**,**17**]. Recently, a two hit hypotheses regarding the occurrence of EPS has been suggested, according to which EPS is developed in two phases: the long duration of PD may induce the occurrence of peritoneal sclerosis and fibrosis (first hit), followed by a second phase represented by inflammation (second hit) [**7**]. The clinical diagnostic is difficult in the early stages, as the symptomatology is unspecific. The clinical elements derive from the alteration of motility and the alteration of the intestinal absorption function and become evident at the same time with the occurrence complications such as the intestinal occlusion. The attempt to identify biomarkers for the diagnostic of EPS has not yielded results until now [**12**]. The decrease of the ultrafiltration capacity, the increase in transporting small solutes as well as the presence of inflammation markers are neither specific nor sensitive to the positive diagnosis of EPS [**1**]. From the imagistic point of view, the computerized type has proven itself useful in supporting the clinical diagnostic by highlighting the thickening of the peritoneum and the intestinal wall, the peritoneal calcification, the loculated fluid collections in the peritoneal cavity and the agglutination of the intestinal loops. Based on these grounds, it is possible to calculate a score, whose high value is associated with a positive diagnostic of EPS [**15**]. After determining the diagnostic, it is generally accepted to discontinue the PD and to transfer the patient on haemodialysis; moreover, the association of an immune-suppressing therapy and the total parenteral nutrition have proven to be useful in the treatment of EPS [**1**]. Nevertheless, the surgical release of the small bowel loops comprised in the encapsulation process is required in advanced cases, but it is associated with high rates of mortality unless performed by surgical centers experienced in the treatment of EPS [**7**]. The long-term prognostic of patients with EPS remains a reserved one; however, it can be improved by making an early diagnostic obtained by means of a high degree of clinical suspicion and thoroughness in its paraclinical confirmation [**1**].

The purpose of this paper is to analyze the clinical characteristics of the patients admitted in our surgery clinic and diagnosed with encapsulating peritoneal sclerosis. 

## Method

The data belonging to patients diagnosed with encapsulating peritoneal sclerosis (EPS) between 2007 and 2014 have been retrospectively analyzed. The patients were admitted at “I. Juvara” Surgery Clinic within “Dr. I. Cantacuzino” Clinical Hospital. 432 surgical interventions related to peritoneal dialysis were performed during this interval: 306 peritoneal access interventions and 124 complications, of which 15 patients with EPS.

## Results

**Fig. 1:**
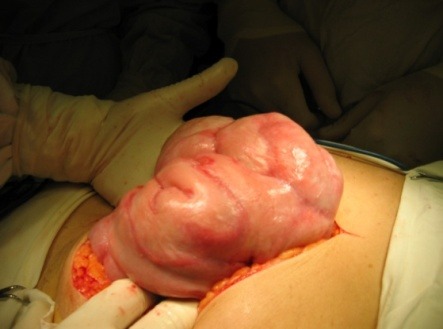
Intraoperative aspect of an advanced EPS - the small bowel loops are agglutinated as in a cocoon.

**Fig. 2:**
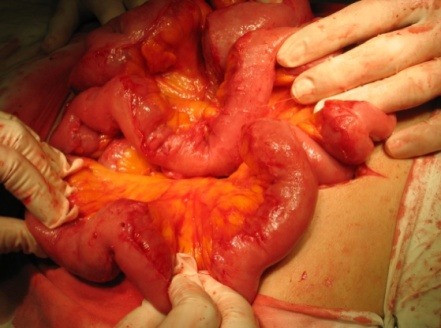
Intraoperative aspect –final aspect after adhesiolysis

**Fig. 3:**
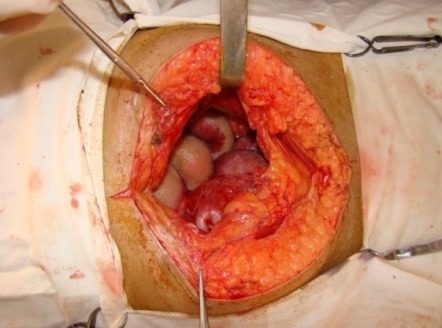
Intraoperative aspect of an incipient encapsulating peritoneal sclerosis (EPS)

In all but two cases, the EPS diagnostic was established at the time of the surgical intervention, addressed to other complication or pathology. Moreover, in 2 of the 15 patients, the diagnostic was established approximately 5 months after PD was discontinued, and, in one of these patients, at the time of extraction of the dialysis catheter. The average age of the patients (8 males and 7 females) was of 59 ± 11.1 years (range 34 to 73), the average duration of dialysis was of 56.53±39.9 months (range 14 to 136). 5 patients had more than 3 episodes of peritonitis as antecedents, 6 patients had between 1 and 3 episodes and, in 2 patients, the number of peritonitis episodes could not be established, one patient was at his first episode at the time of the diagnostic and one patient had no peritonitis at all, but instead suffered from a wound and subcutaneous tunnel infection at the time of the peritoneal access. The average duration of hospitalization was of 7.6 ± 5.3 days. Diabetes mellitus was present in 12 of the 15 patients. The surgical intervention indication was peritonitis refractory to treatment (10 cases), complicated parietal defects (1 case of umbilical hernia and 1 case of incarcerated incisional hernia), bowel obstruction (1 case) and the extraction of catheter due to ultrafiltration failure (2 cases). Intraoperatively, the typical aspect of advanced EPS represented by the thickening of the parietal and visceral peritoneum associated with agglutination of the small bowel loops covering thereof by a fibrous membrane (**Fig. 1**) was identified in 13 cases, in two cases, the intraoperative aspect is of an incipient EPS – parietal and visceral peritoneal thickening without an intestinal obstruction (**Fig. 2**). The surgical intervention consisted of an extraction of the dialysis catheter, washing and drainage of the peritoneal cavity in all patients; in 8 patients it was necessary to perform an adhesiolysis (**Fig. 3**) with a complete release of the small bowel loops and also to resolve parietal defects in the two patients. Peritoneal biopsies were sampled in 11 patients and the histopathology examination revealed the presence of fibrosis and of inflammation infiltration, however, without being able to provide specific elements. The immediate post-operatory evolution was favorable for all but the 3 cases who died. A fourth patient died 2 months after the surgical intervention that has set as a diagnostic the presence of EPS, however, the death could not be linked to it. 

## Discussions

The Encapsulating Peritoneal Sclerosis is a rare complication associated with peritoneal dialysis but with a reserved prognostic when the diagnostic is set in advanced stages. The exact cause of this is not known. Multiple factors may contribute to its occurrence. Among these, the following should be mentioned: the long duration of peritoneal dialysis, repeated episodes of peritonitis, use of acetate-based dialysis solutions or of hypertonic glucose solutions, use of in-line bacterial filters or exposure to certain antiseptic substances, such as chlorhexidine [**9**]. Moreover, a series of factors not linked to PD may contribute to the occurrence of EPS: treatment with β-blocking agents, autoimmune diseases, intraperitoneal chemotherapy, exposure to talc or asbestos, intra-abdominal infections (tuberculosis), genetic factors [**1**,**3**,**4**,**13**]. Even in our series, diabetes mellitus was found in 80% of the cases as Krote el al. mentioned in a Dutch EPS study, and did not find any relation with the development of EPS [**13**]. The reported incidence varies between centers and around the globe, ranging between 0.8 and 19.4% and increasing along with the PD duration [**4**,**5**]. However, the incidence could not be calculated in our study; EPS accounting for 12% of the number of complications that needed a surgical intervention. The average duration of PD was of 4.5 years, in 12 patients (80%), the duration was of over 3 years; only 3 patients (20%) had a PD duration of less than 2 years. Peritoneal remodeling is a process in which the aggression over the mesothelial cells plays an important role. As a response to repeated aggression applied by substances such as the glucose, the mesothelial cells secret cytokines that in turn attract macrophages and fibroblasts [**10**,**11**,**18**]. The sedimentation of fibrin on a denuded mesothelium, as a result of a disrupted repair, leads to peritoneal fibrosis. This process is more accentuated in young patients, who can determine a more difficult onset of EPS modifications [**13**].

Most of the analyzed cases associated a refractory peritonitis at the time of diagnostic (10 patients), however, only 33% had more than 3 episodes of peritonitis in their antecedents. Of the 10 patients with peritonitis, 5 have had positive cultures for fungi (4 candida, 1 aspergillus), 3 had bacterial problems (1 - kelbsiella,1- SA coagulase-positive,1- MRSE staphylococcus coagulase-negative), and in two of the cases, the cultures were sterile. Peritonitis cases are incriminated for the occurrence of EPS, especially such severe episodes or refractory to treatment, and also, the sterile peritonitis episodes may suggest the occurrence of EPS. The frequently incriminated germs are the S. aureus, fungi and/ or Pseudomonas sp. A Japanese study performed by Kawanishi et al. in 2004, showed that 25% of the EPS cases were associated with a bacterial peritonitis [**4**]. Peritonitis with S. aureus represent a major risk factor for EPS because coagulase-positive organisms have an enzymatic activity capable of converting the fibrinogen into fibrin, a main component of inter-visceral adherences [**13**,**20**].

The diagnostic of positive EPS in our case series was established intraoperatively. Although the majority of patients showed a suggestive symptomatology, it could have been attributed to the main impairment for which the surgical indication was prescribed (refractory peritonitis or complicated parietal defects). The symptomatology of EPS on first appearance is often vague and non-specific: nausea, vomiting, fullness, anorexia, weight loss. The symptoms and signs are the results of impairments to the motility and intestinal absorption function due not only to the entrapment of the bowel by the encapsulating process, but also to the destruction of the myenteric plexus by the fibrotic changes [**24**]. Characteristically for EPS is the insidious on set and progressive accentuation of the symptomatology. There are present signs of bowel obstruction in advanced cases which are not hard to identify but the elimination of other causes of disturbed intestinal motility (adhesions, infections, other intra-abdominal pathology) is required. The relationship between peritonitis and EPS is likely complex. Recurrent infections may contribute to the development of EPS and the presence of EPS may be associated with greater predisposition to peritonitis [**1**]. Of the imagistic explorations, the computerized tomography is the most valuable and therefore it is the first recommended investigation [**14**]. Suggestive elements for the establishment of the diagnostic are the peritoneal thickening and calcification, thickening of the intestinal wall, agglutination of bowel loops and loculated ascites [**15**]. The other investigations used are the plain abdominal radiography and ultrasound. The abdominal radiography may find air fluid levels, peritoneal calcifications and dilated small bowel loop, while ultrasonography may reveal a characteristic trilaminar appearance of the intestinal wall, but requires peritoneal fluid in situ. There are no blood or effluent markers specific to the diagnosis of EPS, elevated levels of C-reactive protein, anemia, hypoalbuminemia may be found but are nonspecific [**14**]. The diagnostic of EPS is based on the clinical suspicion primarily confirmed with radiological findings. Laparotomy is the only way to make a definitive diagnostic but it can be associated with considerable morbidity and mortality [**1**,**14**].

It is generally accepted that, after a diagnosis of EPS, PD should be discontinued, and the patient transferred to hemodialysis. Some patients are diagnosed only after the discontinuation of PD and also in some cases with less severe EPS, stopping PD may exacerbate the condition [**14**,**19**,**21**]. In addition, drug therapy with corticosteroids [**4**], tamoxifen [**8**], and immunosuppressants [**22**] have been reported to be beneficial for EPS in a small number of cases. EPS can lead to severe malnutrition, so nutrition support is crucial. Sometimes, total parenteral nutrition forms an integral part of both conservative approach and surgical intervention for a prolonged period of time [**1**]. Surgical enterolysis is considered for severe intestinal obstruction. Mortality after the surgical intervention, which was as high as 50% in the past, can be reduced to 4% in the hands of an experienced surgeon. Poor understanding of EPS characteristics and the utilization of a surgical technique similar to those applied in conventional bowel obstruction involving wide resections and anastomosis may lead to severe complications and death. The basic surgical technique for EPS, reported by Kawanishi et al. consists in a sharp ablation of the capsules and intestinal adhesions. By using this technique, a successfully improvement of the bowel obstruction state was obtained in a series of 50 patients [**7**].

In our series, the catheter was removed and the PD discontinued in all patients except for one patient in whom the catheter was removed 5 months earlier. In 8 of the patients (53.3%) with bowel obstruction sings, adhesiolysis was necessary to release the small bowel from the inflammatory process. Postoperatory evolution was favorable for all but 3 cases (20%) who died. A fourth patient died from a myocardial infarction 2 months after the surgical intervention, so the death cannot be linked to EPS. Between the rest of the three patients, 2 died from severe and prolonged unsolved sepsis which lead to multiple organ failure and one from a severe digestive hemorrhage. 

The overall prognosis of EPS on PD can be poor due to age and associated comorbidities. Reported mortality varies between 26-58% due to different factors, which include different populations, different time periods and the definition of EPS. It was also found that mortality increased with the length of time on PD [**4**,**23**].

## Conclusions

Encapsulated peritoneal sclerosis is a rare condition. The risk of developing EPS is considered extremely low during the first 3 years of PD treatment. There are no predictive and reliable screening tests especially in the early stages of EPS. Diagnosis is based on a high index of suspicion therefore, it is important not to underestimate the clinical symptoms. Computed tomography is a reliable tool for the confirmation of the diagnosis. Surgical techniques are an important treatment option but it is necessary to precisely define the role and timing of surgery in EPS. Collaborative research will be essential if this serious problem facing PD is to be solved.
